# Grip strength and depressive symptoms in Chinese middle-aged and older adults: the mediating effects of cognitive function

**DOI:** 10.3389/fnagi.2024.1455546

**Published:** 2024-10-09

**Authors:** Xinzheng Wang, Lifei Wu, Huifen Zhou, Jiandong He

**Affiliations:** ^1^Department of Physical Education, Zhejiang Chinese Medical University, Hangzhou, China; ^2^College of Basic Medical Sciences, Zhejiang Chinese Medical University, Hangzhou, China

**Keywords:** grip strength, cognitive function, depression symptoms, mediation analysis, older Chinese adults

## Abstract

**Objective:**

This study investigates the associations and mediating pathways between grip strength, cognitive function, and depression in middle-aged and elderly individuals in China.

**Methods:**

Utilizing data from the 2011 China Health and Retirement Longitudinal Study (CHARLS), we employed logistic regression and mediation analysis to examine the relationships and mediating factors between grip strength, cognitive function, and depression, while adjusting for potential confounders.

**Results:**

The study included 6,841 participants, of whom 1,734 (25.35%) exhibited symptoms of depression. Our findings indicate that weak grip strength is significantly associated with an increased risk of depression (OR: 1.57, 95% CI: 1.32–1.87) among the middle-aged and elderly population. Conversely, good cognitive function was found to be protective against depression (OR: 0.94, 95% CI: 0.93–0.95). Grip strength indirectly affected depression through cognitive function, accounting for 9.4% of the total effect (*β* = −0.008, 95% CI [−0.013, −0.004]). This mediating effect was 23.8% in men (*β* = −0.013, 95% CI [−0.020, −0.007]); and 23.2% in those aged 60 years and over (*β* = −0.015, 95% CI [−0.022, −0.009]).

**Conclusion:**

This study highlights that weak grip strength increases risk of depressive symptoms, and adequate cognitive function can mitigate the association between weak grip strength and an increased risk of depression among middle-aged and elderly individuals in China. Psychological care for elder adults with weak grip strength and poor cognitive function should be strengthened.

## Introduction

Depression is a significant mental health concern and is increasingly recognized as a leading cause of disabling mental disorders worldwide (GBD 2019 Diseases and Injuries Collaborators, 2020). The World Health Organization has predicted that by 2030, depression will become one of the most impactful global health issues (Dwyer et al., 2020). In China, which is undergoing rapid population aging, more than 54 million individuals are affected by depression, as reported by World Health Organization (2017). The prevalence of depression is particularly high among the middle-aged and elderly, those aged 45 and above (Lei et al., 2014), with a detection rate of 20.6% in the population over 60 years old (Wang et al., 2023). Moreover, the incidence of depression increases with age (Yang, 2007). Depression not only significantly impairs the quality of life of affected individuals (Ji et al., 2023) but also increases the risks of suicide (Ruggieri, 2020), type 2 diabetes (Semenkovich et al., 2015), cardiovascular diseases, and overall mortality (Krittanawong et al., 2023). Therefore, understanding the etiology and pathophysiology of depression, particularly in the middle-aged and elderly population in China, is of great public health importance. However, our understanding of depression is still incomplete, and more effective treatment methods are needed (Malhi and Mann, 2018). Identifying risk factors for depression and implementing effective interventions and management strategies are therefore crucial.

Among various factors associated with depression, the impact of weak grip strength on depressive symptoms has recently gained attention (Fukumori et al., 2015; Lee et al., 2018; Marques et al., 2020). However, research data on the correlation between grip strength and depression in elderly populations are limited, and the nature of this association remains unclear. Studies by Zhang et al. (2023) and Brooks et al. (2018), using data from the China Health and Retirement Longitudinal Study (CHARLS) and the National Health and Nutrition Examination Survey (NHANES), suggest a negative correlation between grip strength and depression, after adjusting for confounding factors. Conversely, Zhang et al. (2021) found in a cross-sectional study that the risk of depression decreased with increasing grip strength, but the association was not significant when handgrip strength (HGS) exceeded 36.5 kg. In contrast, studies by Stessman et al. (2017) and Taekema et al. (2010) observed a weak or non-existent predictive role of declining grip strength in the onset of depression in older adults. This suggests that the relationship between muscle strength and depression may be influenced by the psychological adaptation process during aging. It is therefore essential to understand the association between grip strength levels and depression among middle-aged and elderly individuals in China to inform the development of intervention measures.

Additionally, recent evidence suggests a strong association between cognitive function and depressive symptoms, with better cognitive states in older individuals linked to good emotional states (Kaczorowska et al., 2024; Rock et al., 2014). Studies by Ferri et al. (2021) and Christensen et al. (2018) have shown that cognitive impairments in executive function, memory, and attention are common in individuals with depression, and that alleviating these cognitive deficits can contribute to improving depression symptoms. Moreover, research has shown a connection between grip strength and cognitive function (Yang et al., 2022). A study in rural China found that lower handgrip strength (HGS) and HGS asymmetry are independently associated with lower cognitive function (Feng et al., 2023), and studies by Firth et al. (2018) indicate a significant correlation between higher grip strength in older individuals and better cognitive performance (Jin et al., 2022). However, the association between grip strength and cognitive function in the elderly has not reached a consensus. Cui et al. (2021) reported that in older adults, grip strength is positively correlated with recall and memory performance but not with general cognitive function. In contrast, some studies have found no relationship between grip strength and cognitive dysfunction (Sibbett et al., 2018; Doi et al., 2019). This discrepancy may be due to the complexity of motor function and needs further investigation. Our study aims to explore the potential mediating pathways between grip strength, cognitive function, and depression in middle-aged and elderly Chinese adults using data from the China Health and Retirement Longitudinal Study (CHARLS).

## Materials and methods

### Data source

This study is based on data from the China Health and Retirement Longitudinal Survey (CHARLS). CHARLS conducted a comprehensive baseline survey across the nation utilizing a probability-proportional-to-size (PPS) sampling method. This extensive survey gathered a wide array of information, including respondents’ basic demographic details, their family’s data, and insights into their education, employment, income, marital status, and health status. In addition, CHARLS incorporated an extensive set of 13 physical measurements and also undertook blood sample collection. Each stage of this survey was subject to stringent quality control measures, establishing CHARLS as a vital and authoritative source for investigating the array of health issues facing China’s elderly population. The data from this extensive survey is publicly available and can be accessed at http://charls.pku.edu.cn. The CHARLS project was conducted with full approval from the Peking University Biomedical Ethics Committee (IRB0000001052-11015), ensuring ethical compliance, and informed consent was duly obtained from all individuals who participated in the survey (Zhao et al., 2014).

**Figure 1:**
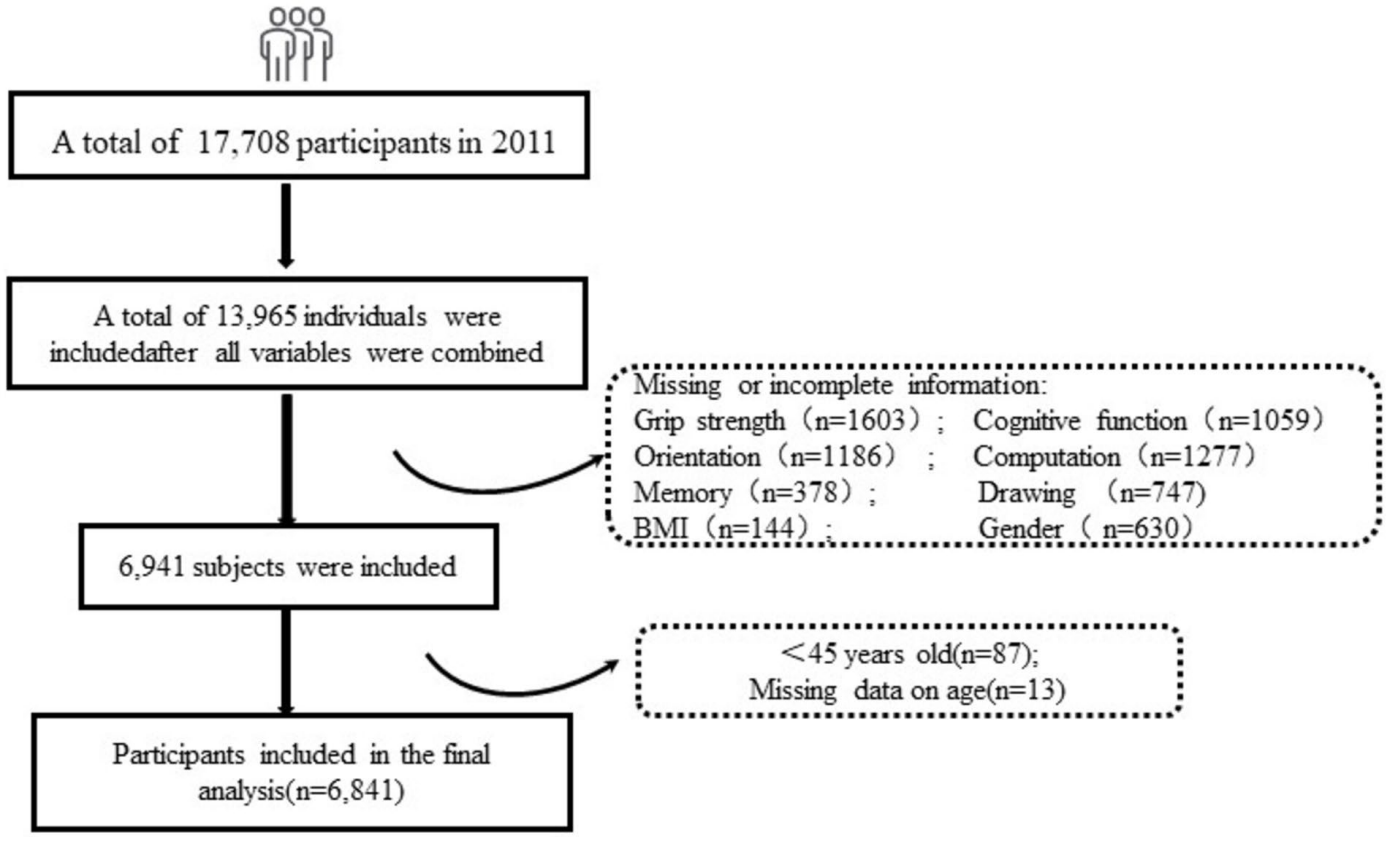
Flow chart of the selection of study participants.

For this particular study, we used the data from the 2011 CHARLS survey, which originally consisted of 17,708 participants. We had to exclude some participants from our analysis due to missing data in key areas such as grip strength, cognitive function (which included aspects like orientation, memory, calculation, and drawing), and depression scores. Additionally, we also excluded participants who had missing values in covariate data crucial for our sample analysis. After applying these exclusion criteria, our study eventually included data from 6,841 participants, which was sufficient for a robust analysis (Figure 1).

### Variable selection

#### Demographic characteristics

The demographic characteristics of the survey participants were meticulously extracted from two specific modules of the CHARLS follow-up questionnaire: the “Basic Information” module and the “Family” module. This extraction process yielded vital information such as the participants’ gender, age, marital status, and their level of educational attainment.

#### Grip strength

The measurement of grip strength was conducted using a specialized isometric dynamometer (Yuejian TM WL-1000, Nantong, China) (Bao et al., 2022). Participants were instructed to perform this test while standing, using either their dominant or non-dominant hand, based on their preference. They were required to keep their elbow positioned at a precise right angle (90°) and then exert force by squeezing the handle of the dynamometer for several seconds. This process was repeated to allow two measurements for both the right and left hands, and the highest value recorded in kilograms was used for analysis. We then categorized grip strength based on gender and body mass index (Mak et al., 2023), employing a binary scale where 0 indicated a normal grip strength level, and 1 indicated a comparatively weak grip strength (Supplementary Table S1).

#### Cognitive function

Cognitive function was evaluated based on the methodology employed in the American Health and Retirement Study (HRS) (Crimmins et al., 2011). This comprehensive assessment involved evaluating four key dimensions of cognitive function: orientation, calculation, memory, and drawing ability. The orientation assessment involved questions about the current year, month, day, day of the week, and the season, with each correct answer scoring 1 point, making a total possible score of 5 points. The calculation dimension required participants to perform serial subtractions, starting from 100 and subtracting 7 each time, up to five iterations. Each successful subtraction earned them 1 point. The memory assessment involved presenting 10 random words to each participant, with their immediate recall capacity being evaluated based on the number of words they could immediately recollect. Following this, the participants completed the depression scale survey, after which their calculation and drawing abilities were tested. An assessment of delayed word recall was then conducted. The total memory score was a combination of both immediate and delayed word recall, with each correctly recalled word earning 1 point. The drawing test involved presenting participants with a picture of two overlapping pentagons, which they were then asked to replicate accurately. Successful replication earned the participant 1 point. The total score for cognitive function was thus calculated as the sum of scores from these four dimensions: orientation (5 points), calculation (5 points), memory (20 points), and drawing (1 point), giving a total possible score of 31 points. The scoring criteria followed for this assessment were aligned with the methodology used in the American Health and Retirement Study (Cao et al., 2021).

#### Depression symptoms

The assessment of depression was carried out using the 10-item Center for Epidemiologic Studies Depression Scale (CES-D). Each item on this scale was rated on a frequency scale ranging from 0 to 3 (Gabriel et al., 2021). The scale’s specifics were as follows: 0 was used to indicate rare or no occurrence of a symptom (less than 1 day per week), 1 indicated the symptom occurred some or a little of the time (1–2 days per week), 2 was used for occasions or a moderate amount of the time (3–4 days per week), and 3 indicated the symptom was present all of the time (5–7 days per week). On this scale, the occurrence of positive emotions or behaviors was scored in a reverse manner. The scores of all 10 items were summed to obtain a total score, which ranged from 0 to 30, with higher scores indicating more severe symptoms of depression. Individuals who had a CES-D score of 10 or higher were considered to be participants with depressive symptoms.

#### Covariates

This study included a range of additional covariates, such as age, follow-up time, education level, smoking habits, alcohol consumption, body mass index (BMI), hypertension, fall-related injuries, lipid abnormalities, diabetes or elevated blood sugar, cancer or malignant tumors, heart disease, stroke, nearsightedness and farsightedness, injuries, hearing issues, memory-related disorders, etc. Categories like hearing, vision, and life satisfaction were divided into three levels: “poor, moderate, good.” Marital status was categorized as married (living with a spouse) or unmarried (separated, divorced, widowed, or never married). Permanent residence was classified as urban or rural. Other variables were dichotomized into a simple “yes or no” format.

### Statistical analysis

The characteristics of the sample were thoroughly measured and subjected to a descriptive analysis. To identify differences between groups with and without depressive symptoms at baseline, variance *t*-tests were employed for numerical variables, and chi-square tests were used for categorical variables. The variables are presented in different formats for clarity: continuous variables are shown as medians, with the interquartile range (25–75%) in parentheses, while categorical variables are expressed as counts (percentages). To determine the associations between grip strength groups, cognitive function, and depressive symptoms, both binary logistic regression and multiple linear regression analyses were employed. These analyses produced effect estimates and 95% confidence intervals (CI). Three distinct models were introduced for this purpose: Model 1, which was unadjusted; Model 2, which was adjusted for Gender, Age, BMI; and Model 3, which was adjusted for the variables in Model 2, as well as additional factors like Education, Permanent address, Marital status, Hypertension, Dyslipidemia, Diabetes, Psychiatric problems, Smoking, and Alcohol consumption. Additionally, Pearson correlation analysis was conducted to examine the relationships between the variables involved in this study.

Mediation analysis was a significant component of this module, and it was performed using the Mediation package available in R. The bootstrap method was employed to estimate confidence intervals for the mediation effects. The significance of these mediation effects was determined based on whether the confidence interval included 0 (indicating non-significance) or did not include 0 (indicating significance). Furthermore, to assess the stability of results and examine gender and age differences in the associations between grip strength, cognitive function, and depressive symptoms, subgroup analyses of the binary logistic regression models were conducted, with these analyses being segmented by gender (male and female) and age groups (<60 and ≥ 60). For all analyses conducted in this study, a significance level of *p* < 0.05 was considered statistically significant. The analytical processes were carried out using two software programs: SPSS 26.0 and R (version 4.2.2).

## Results

### Baseline characteristics analysis of the sample

Table 1 showcases the baseline characteristics of the participants from the China Health and Retirement Longitudinal Study (CHARLS) database. Among the 6,841 subjects included in this analysis, 1,734 individuals (25.35%) exhibited depressive symptoms. A total of 6,077 (88.8%) participants were classified in the normal grip strength group, while 764 (11.2%) fell into the weak grip strength group. The analysis revealed that participants who were female, lived in urban areas, were unmarried (widowed/separated/single), had lower educational levels, poor hearing, and poor vision, and belonged to the weak grip strength group, had a heightened risk of developing depressive symptoms (*p* < 0.001). Moreover, individuals with hypertension, diabetes, chronic lung diseases, kidney disease, digestive disease, and psychiatric problems also displayed an increased risk of depression (*p* < 0.05).

**Table 1:** Characteristics of study participants stratified by depressive symptoms.

Variables	Total	No-Depression	Depression	*p*
	N = 6,841	N = 5,107	N = 1734	
Age	59.00 [52.00, 65.00]	59.00 [52.00, 65.00]	58.50 [51.00, 65.00]	0.545
Gender (%)				<0.001
Male	3,682 (53.8)	2,961 (58.0)	721 (41.6)	
Female	3,159 (46.2)	2,146 (42.0)	1,013 (58.4)	
Grip strength (%)				<0.001
Normal	6,077 (88.8)	4,585 (89.8)	1,492 (86.0)	
Weak	764 (11.2)	522 (10.2)	242 (14.0)	
Education (%)				<0.001
Junior high and below	5,791 (84.7)	4,246 (83.1)	1,545 (89.1)	
high school and above	1,050 (15.3)	861 (16.9)	189 (10.9)	
Permanent address (%)				<0.001
Urban	5,073 (74.2)	3,724 (72.9)	1,349 (77.8)	
Rural	1768 (25.8)	1,383 (27.1)	385 (22.2)	
Marital status (%)				<0.001
Married	721 (10.5)	468 (9.2)	253 (14.6)	
Unmarried	6,120 (89.5)	4,639 (90.8)	1,481 (85.4)	
Smoking (%)				<0.001
No	3,842 (56.2)	2,757 (54.0)	1,085 (62.6)	
Yes	2,999 (43.8)	2,350 (46.0)	649 (37.4)	
Alcohol consumption (%)				<0.001
No	4,363 (63.8)	3,152 (61.7)	1,211 (69.8)	
Yes	2,478 (36.2)	1955 (38.3)	523 (30.2)	
Hypertension (%)	1,694 (24.8)	1,215 (23.8)	479 (27.6)	0.002
Dyslipidemia (%)	706 (10.3)	519 (10.2)	187 (10.8)	0.49
Diabetes (%)	417 (6.1)	287 (5.6)	130 (7.5)	0.006
Cancer (%)	60 (0.9)	40 (0.8)	20 (1.2)	0.2
Chronic lung diseases (%)	676 (9.9)	441 (8.6)	235 (13.6)	<0.001
Liver disease (%)	280 (4.1)	193 (3.8)	87 (5.0)	0.029
Kidney disease (%)	417 (6.1)	273 (5.4)	144 (8.3)	<0.001
Digestive disease (%)	1,478 (21.6)	971 (19.0)	507 (29.3)	<0.001
Psychiatric problems (%)	65 (1.0)	30 (0.6)	35 (2.0)	<0.001
Memory related disease (%)	69 (1.0)	43 (0.8)	26 (1.5)	0.026
Arthritis or rheumatism (%)	2,115 (30.9)	1,387 (27.2)	728 (42.0)	<0.001
Asthma (%)	253 (3.7)	156 (3.1)	97 (5.6)	<0.001
Nighttime sleep duration	6.00 [5.00, 7.50]	6.00 [5.00, 7.50]	6.00 [5.00, 7.00]	0.14
Poor sleep quality (%)				<0.001
Rarely or none of the time	3,597 (52.6)	3,247 (63.6)	350 (20.2)	
Some or a little of the time	1,145 (16.7)	885 (17.3)	260 (15.0)	
Occasionally or a moderate amount of the time	917 (13.4)	506 (9.9)	411 (23.7)	
Most or all of the time	1,182 (17.3)	469 (9.2)	713 (41.1)	
Hearing (%)				<0.001
Good	3,292 (48.1)	2,605 (51.0)	687 (39.6)	
Fair	2,871 (42.0)	2083 (40.8)	788 (45.4)	
Poor	678 (9.9)	419 (8.2)	259 (14.9)	
Life satisfaction (%)				<0.001
Good	5,872 (85.8)	4,568 (89.4)	1,304 (75.2)	
Fair	836 (12.2)	482 (9.4)	354 (20.4)	
Poor	133 (1.9)	57 (1.1)	76 (4.4)	
Waistline	85.30 [78.40, 92.20]	86.00 [78.60, 92.60]	84.60 [78.00, 92.00]	0.003
BMI (median [IQR])	23.44 [21.15, 25.99]	23.46 [21.20, 26.03]	23.32 [20.96, 25.89]	0.089
Memory (median [IQR])	8.00 [5.00, 10.00]	8.00 [5.00, 10.00]	7.00 [5.00, 9.00]	<0.001
Orientation (median [IQR])	5.00 [4.00, 5.00]	5.00 [4.00, 5.00]	4.00 [3.00, 5.00]	<0.001
Computation (median [IQR])	5.00 [1.00, 5.00]	5.00 [2.00, 5.00]	3.00 [1.00, 5.00]	<0.001
Drawing (%)				<0.001
0 points	1,683 (24.6)	1,140 (22.3)	543 (31.3)	
1 point	5,158 (75.4)	3,967 (77.7)	1,191 (68.7)	
Vision (%)				<0.001
Good	3,588 (52.7)	2,815 (55.4)	773 (44.7)	
Fair	2,693 (39.5)	1973 (38.8)	720 (41.6)	
Poor	529 (7.8)	292 (5.7)	237 (13.7)	
Cognitive function (median [IQR])	16.00 [13.00, 19.00]	17.00 [13.00, 20.00]	15.00 [12.00, 18.00]	<0.001

### Associations of grip strength and cognitive function with depression

Table 2 delineates the relationship between grip strength levels, cognitive function, and depression. After adjusting for various confounders, it was found that older adults in the weak grip strength group were more likely to develop depressive symptoms compared to those in the normal grip strength group (OR: 1.57, 95% CI: 1.32–1.87). Enhanced cognitive function (OR: 0.94, 95% CI: 0.93–0.95), along with better orientation (OR: 0.87, 95% CI: 0.83–0.93), memory (OR: 0.94, 95% CI: 0.92–0.95), computation (OR: 0.90, 95% CI: 0.87–0.93), and drawing abilities (OR: 0.93, 95% CI: 0.92–0.94), were identified as protective factors against depression.

**Table 2:** Associations of grip strength and cognitive function with depression.

Variables	Model 1	Model 2	Model 3	*p*-value
Grip strength				<0.001
Normal	1	1	1	
Weak	1.42(1.21–1.68)	1.61(1.35–1.91)	1.57(1.32–1.87)	
Cognitive function	0.93(0.92–0.94)	0.93(0.92–0.94)	0.94(0.93–0.95)	<0.001
Orientation	0.84(0.56–0.86)	0.85(0.81–0.90)	0.87(0.83–0.93)	<0.001
Memory	0.93(0.92–0.94)	0.93(0.91–0.94)	0.94(0.92–0.95)	<0.001
Computation	0.87(0.84–0.89)	0.89(0.86–0.91)	0.90(0.87–0.93)	<0.001
Drawing				<0.001
0 points	1	1	1	
1 point	0.63(0.55–0.71)	0.68(0.60–0.77)	0.93(0.92–0.94)	

Model 1, unadjusted; Model 2, adjusted for Gender, Age, BMI; Model 3, adjusted for variables in Model 2 as well as Education, Permanent address, Marital status, Hypertension, Dyslipidemia, Diabetes, Psychiatric problems, Smoking, Alcohol consumption.

### The bivariate correlations for all variables

Table 3 presents the bivariate correlations for all variables. It was observed that grip strength and cognitive function (including memory, drawing, orientation, computation) were negatively associated with depression. On the other hand, grip strength showed a positive association with depression, justifying further moderated mediation analysis.

**Table 3:** Correlation for the main variables.

Variable	1	2	3	4	5	6	7
1. Grip strength	–						
2. Depression	−0.148^***^	–					
3. Cognitive function	0.220^***^	−0.133^***^	–				
4. Memory	0.148^***^	−0.091^***^	0.874^***^	–			
5. Drawing	0.194^***^	−0.073^***^	0.422^***^	0.239^***^	–		
6. Orientation	0.161^***^	−0.085^***^	0.555^***^	0.297^***^	0.310^***^	–	
7. Computation	0.175^***^	−0.120^***^	0.628^***^	0.247^***^	0.269^***^	0.280^***^	–

^***^p<0.001.

### Mediating effects of cognitive function in the association between grip strength and depression

The results illustrated the impact of grip strength on depression through cognitive function, while controlling for 11 covariates potentially associated with the study variables (Table 4). Grip strength level was a significant negative predictor of depression (*β* = −0.088, *p* < 0.001), and a significant positive predictor of cognitive function (β = 0.220, p < 0.001). Additionally, a negative correlation was found between cognitive function and depression (β = −0.133, p < 0.001). The indirect influence of grip strength on depression through cognitive function was also revealed (*β* = −0.008, 95% CI [−0.013, −0.004]), accounting for 9.4% of the effect.

**Table 4:** The moderated mediating effect of grip strength class on depression by cognitive function.

Independent variable	Mediator	Total effect, coefficient (95% CI)	Indirect effect, coefficient (95% CI)	Direct effect, coefficient (95% CI)	Proportion mediated, % (95% CI)
Grip strength class	Drawing	−0.087 (−0.126, –0.05)***	−0.000 (−0.004, 0.001)*	−0.086 (−0.125, −0.052)***	0.3 (−1.3, 4.4)
	Memory	−0.088 (−0.127, −0.054)***	−0.005 (−0.008, −0.002)***	−0.083 (−0.121, −0.050)***	5.6 (1.9, 11.6)
	Orientation	−0.087 (−0.126, −0.053)***	−0.003 (−0.006, −0.001)**	−0.084 (−0.123, −0.051)***	3.6 (1.2, 7.7)
	Computation	−0.087 (−0.126, −0.053)***	−0.004 (−0.007, −0.001)**	−0.084 (−0.123, −0.050)***	4.2 (0.9, 8.9)
	Cognitive function	−0.088 (−0.127, −0.054)***	−0.008 (−0.013, −0.004)***	−0.080 (−0.118, −0.046)***	9.4 (4.6, 17.4)

The mediation analyses were adjusted for age, BMI, gender, education, permanent address, marital status, hypertension, dyslipidemia, diabetes, smoking and alcohol consumption. CI, Confidence interval. *p* value; ***p<0.001, **p < 0.05, *p > 0.05.

### Sensitivity and subgroup analyses

The sensitivity analysis was enhanced by conducting subgroup analyses stratified by gender and age. These analyses disclosed no significant age or gender differences in the relationship between weak grip strength, cognitive function, and the incidence of depression. Cognitive function mediates the relationship between grip strength and the prevalence of depression, and the neutralizing effect is more pronounced in men and people aged 60 and over (Supplementary Tables S2, S3).

## Discussion

This study represents a pioneering exploration into the mediating role of cognitive function in the relationship between grip strength and the incidence of depression among middle-aged and elderly individuals in China. Our research findings underscore that weak grip strength, after accounting for potential confounding factors, is a notable risk factor for depression within this demographic group (OR: 1.57, 95% CI: 1.32–1.87). In contrast, an elevated level of cognitive function (OR: 0.94, 95% CI: 0.93–0.95) emerges as a protective factor against depression among the middle-aged and elderly population in China. Interestingly, our results indicate that cognitive function partially mediates the relationship between grip strength and the incidence of depression.

The utility of grip strength as a straightforward, economical risk stratification tool is well-recognized, particularly in clinical settings. Its simplicity, portability, and low cost make it an attractive tool for assessing individual health risks (Zare et al., 2023). Previous research into grip strength and depression has indicated that weak grip strength might be a risk factor for depression in middle-aged and elderly individuals (Muhammad and Maurya, 2022; López-Bueno et al., 2023). These studies have suggested a dose–response relationship between grip strength and depression risk, highlighting a negative correlation between grip strength and the risk of depression. Our study aligns with these findings, as we discovered that individuals with weaker grip strength, after adjusting for potential confounding factors, have a 1.45 times higher odds of developing depression (Ashdown-Franks et al., 2019). Elderly individuals in the lowest quartile of muscle strength are more prone to experiencing symptoms of depression and suicidal thoughts (Han et al., 2019). Our study extends these insights by stratifying grip strength based on body mass index (BMI) and identifying weak grip strength levels under different BMI conditions (Mak et al., 2023). We delved into analyzing the relationship between the weak grip strength group and the incidence of depression, suggesting that weak grip strength levels can be a useful indicator for monitoring depression in the middle-aged and elderly population. Our findings advocate for primary healthcare providers to regularly assess grip strength levels in this demographic, especially those with weak grip strength, and to implement appropriate interventions, such as scientific physical exercise or resistance training, to alleviate depression.

The association between cognition and grip strength is also a critical aspect of our findings. Epidemiological studies have established that weaker grip strength is linked to cognitive decline, an increased risk of mental illnesses, and dementia. For example, an analysis involving 190,406 adult participants from the UK Biobank revealed a correlation between hand grip strength and measures of neurocognitive health in both males and females (Duchowny et al., 2022). In populations with depression, cognitive tasks like reaction time and working memory have shown associations with maximum grip strength, influencing the risk of dementia (Firth et al., 2018). The reciprocal relationship between grip strength and cognition is also evident in some studies suggesting that muscle strength deficits lead to cognitive decline (Taekema et al., 2010; McGough et al., 2013). However, this perspective is challenged by conflicting findings in other studies (Abellan van Kan et al., 2013; Ukegbu et al., 2014; Gallucci et al., 2013) that propose individuals with better cognitive abilities exhibit superior handgrip strength (HGS) values compared to those with poorer cognitive abilities. There is significant evidence indicating an association between cognitive changes and reduced grip strength (HGS) (Silva and Menezes, 2016). Cognitive impairments affect the muscle strength of older individuals, influencing their functional capacity and subsequent dependency. A decline in inhibitory function and grip strength is evident in middle-aged individuals, showing a significant correlation (Adamo et al., 2020). A recent study involving 5,995 Korean participants confirmed a bidirectional relationship between grip strength and cognitive function, suggesting a common pathway between these two structures (Kim et al., 2019). This suggests that grip strength can be a supplementary measure for assessing cognitive abilities in older adults.

Furthermore, our study indicates an increase in the likelihood of depression with cognitive decline, with individuals experiencing cognitive decline at an elevated risk of progressing to mild cognitive impairment (MCI) and dementia (Shimada et al., 2014). Numerous longitudinal studies have identified a decline in cognitive abilities among elderly individuals with symptoms of depression or diagnosed depression (Guan et al., 2020; Dotson et al., 2020). The level of cognitive functioning not only influences the severity of depressive symptoms but also impacts the types of symptoms reported (Brailean et al., 2016). Research exploring broader cognitive indicators has encompassed areas such as memory, executive function, reasoning, and processing speed (Jiang et al., 2022). The severity of depressive symptoms is often associated with poorer cognitive performance in neuropsychological tests, revealing specific domain deficits in memory, attention, executive function, and processing speed (Jamieson et al., 2019). The relationship between depression, brain abnormalities, and cognitive decline is dynamic over time, necessitating an analysis of cognitive decline across various domains to investigate subtle early changes related to mental states. These factors may be intricately linked to the complexity of neural and motor functions.

Importantly, our study indicates that cognitive function acts as an intermediary in the relationship between weak grip strength and the likelihood of depression. In our analysis, mediation analysis revealed a significant pathway involving grip strength levels, cognitive function, and depressive status. Further subdivision of cognitive function indicators for mediation analysis revealed that, apart from drawing ability, memory, orientation, and calculation—three cognitive abilities—demonstrated significant mediation effects on the relationship between grip strength and depressive symptoms in middle-aged and elderly individuals (*p* < 0.001). This finding offers new evidence in the existing literature, suggesting that grip strength may positively impact subsequent depression through the cognitive abilities of older adults, including memory, orientation, and calculation. Subgroup analyses in the present study showed that this mediating effect was more pronounced in men as well as in the elderly population aged 60 years and older, which could help guide better clinical diagnosis and management of depression in the context of concomitant low grip strength and cognitive function. Although the mechanisms supporting the impact of cognitive function on the association between grip strength and depressive symptoms are not yet fully understood, several hypotheses have been proposed, both biologically and psychologically. One plausible mechanism linking grip strength to depression involves low-grade inflammation, a condition prevalent in approximately one-fourth of individuals with depression, with over half of these individuals exhibiting mild elevations in C-reactive protein levels (Osimo et al., 2019; Kunutsor et al., 2022). In a study, inflammatory factors (plasma interleukin (IL)-6 and C-reactive protein (CRP)) were shown to be associated with midlife cognition through changes in brain morphology (Marsland et al., 2015). Physical exercise or resistance training, known to increase muscle strength and reduce systemic inflammation, can also improve cognitive function, thereby contributing to the alleviation of depressive symptoms (Verhoeven et al., 2016). Another crucial factor that may influence the relationship between depression and cognition is age-related structural changes in the brain, particularly hippocampal volume loss. Older individuals with depression and reduced hippocampal volume are at a greater risk of cognitive decline (Baksh et al., 2021). Brain-derived neurotrophic factor (BDNF) plays a role in regulating hippocampal plasticity, and its deficiency is implicated in the pathophysiology of depression. BDNF is considered essential for maintaining hippocampal integrity and cognition (Kanellopoulos et al., 2011). Depression is associated with low levels of brain-derived neurotrophic factor (BDNF), which is crucial for emotional processing, memory, and learning (Brunoni et al., 2008). Evidence suggests that the lack of BDNF plays a significant role in the pathophysiology of depression, and exercise-induced increases in BDNF can improve hippocampal atrophy, consequently enhancing cognitive functions such as memory (Erickson et al., 2012), thus supporting the notion of reducing depression. Beyond the impact of exercise on improving inflammation and neurotrophic factors, in psychological terms, exercise may contribute to a sense of mental well-being. Various psychosocial factors may also influence susceptibility to illness. For instance, a robust physique may be associated with reduced psychological distress (i.e., stress and negative impacts) and better mental health (i.e., optimism and self-esteem) (Rodriguez-Ayllon et al., 2018). Individuals engaged in regular exercise are more likely to foster supportive social relationships, potentially reducing the severity of depression (Hallgren et al., 2017). Therefore, comprehensive research is needed in the future to understand how different factors mediate the relationship between grip strength and depression.

Our research findings may have potential theoretical implications for attenuating and improving depressive symptoms in middle-aged and elderly individuals. Weak grip strength levels can serve as an indicator for monitoring depression susceptibility in this population. Primary healthcare practitioners should regularly assess grip strength levels in middle-aged and elderly individuals, with particular attention to those with weak grip strength. Grip strength can be enhanced through physical exercise or resistance training (Khodadad Kashi et al., 2023). Simultaneously, cognitive functions can be improved through activities such as exercise games (Rosenberg et al., 2010), meditation (Nash and Newberg, 2013), music (Hars et al., 2014), and dance therapy (Murrock and Graor, 2014). These interventions aim to mitigate the relationship between low grip strength levels and the risk of depression.

### Limitations

While this study draws on a nationally representative sample of middle-aged and elderly individuals in China, providing novel insights into the moderating role of cognitive function in the relationship between grip strength and depressive symptoms, it is not without its limitations. Firstly, the cross-sectional nature of this study precludes the ability to infer causal or bidirectional relationships among grip strength, cognition, and depression. This limitation is significant as it restricts the understanding of the temporal sequence of these associations. Secondly, the reliance on self-reported data for many variables, including sociodemographic and health-related factors, introduces the potential for recall bias. Participants’ recollections may not always be accurate, which could affect the reliability of the findings. Lastly, while the study made efforts to adjust for various potential confounders, there remains the possibility that other unmeasured factors, such as dietary patterns (Marx et al., 2021), levels of inflammation (Verhoeven et al., 2016), and additional indicators, might have influenced the results. Future research endeavors should consider a more comprehensive approach, possibly incorporating longitudinal designs, to better understand the intricate relationships between depression and its related risk factors.

## Conclusion

This cross-sectional study, utilizing data from the China Health and Retirement Longitudinal Study (CHARLS), found evidence that weak grip strength is associated with an increased risk of depressive symptoms among middle-aged and older adults. Importantly, the study also finds that higher cognitive function may mitigate this association, particularly in older men. These findings underscore a potentially important role for grip strength measurement in the assessment of depression risk in older adults. Targeted interventions, possibly focusing on enhancing grip strength and cognitive function, should be explored as they may help in reducing the risk and severity of depressive symptoms in middle-aged and older adults.

## Data Availability

The raw data supporting the conclusions of this article will be made available by the authors, without undue reservation.
