# Supplementary material for: Grip strength and depressive symptoms in Chinese middle-aged and older adults: the mediating effects of cognitive function
Source: Front Aging Neurosci. 2024 Oct 9;16:1455546. doi: 10.3389/fnagi.2024.1455546 (PMC11497465; doi:10.3389/fnagi.2024.1455546)
Supplement: Supplementary file 1 [file Table_1.docx]

| Measured grip strength (maximum value of either hand), stratified by sex and body mass index (BMI).  1 = weak; 0 = normal | Women & BMI ≤23: 1 = grip strength ≤17 kg |
| --- | --- |
|  | Women & BMI >23–26: 1 = grip strength ≤17.3 kg |
|  | Women & BMI >26–29: 1 = grip strength ≤18 kg |
|  | Women & BMI >29: 1 = grip strength ≤21 kg |
|  | Men & BMI ≤24: 1 = grip strength ≤29 kg |
|  | Men & BMI >24–26: 1 = grip strength ≤30 kg |
|  | Men & BMI >26–28: 1 = grip strength ≤30 kg |
|  | Men & BMI >28: 1 = grip strength ≤32 kg |

Supplementary File 1: Table S1.
