# Supplementary material for: Grip strength and depressive symptoms in Chinese middle-aged and older adults: the mediating effects of cognitive function
Source: Front Aging Neurosci. 2024 Oct 9;16:1455546. doi: 10.3389/fnagi.2024.1455546 (PMC11497465; doi:10.3389/fnagi.2024.1455546)
Supplement: Supplementary file 2 [file Table_2.docx]

Table S2 Subgroup analysis for grip strength, Cognitive Function and risk for depression

| Variables | Group | Model1 | Model2 | P |
| --- | --- | --- | --- | --- |
| Grip strength  Normal |  |  |  | 1 (Reference) |
| Grip strength  Weak | ＜60 | 1.90 (1.43 - 2.53) | 1.91 (1.43 - 2.56) | ＜0.001 |
|  | ≥60 | 1.29 (1.04 - 1.58) | 1.42 (1.14 - 1.76) | ＜0.001 |
|  | Female | 1.42 (1.21 - 1.68) | 1.83 (1.40 - 2.38) | ＜0.001 |
|  | Male | 1.42 (1.21 - 1.68) | 1.40 (1.10 - 1.79) | ＜0.001 |
|  |  |  |  |  |
| Cognitive Function | ＜60 | 0.94 (0.92 - 0.95) | 0.95 (0.93 - 0.96) | ＜0.001 |
|  | ≥60 | 0.93 (0.91 - 0.95) | 0.95 (0.93 - 0.97) | ＜0.001 |
|  | Female | 0.95 (0.94 - 0.97) | 0.96 (0.94 - 0.97) | ＜0.001 |
|  | Male | 0.92 (0.91 - 0.94) | 0.92 (0.90 - 0.94) | ＜0.001 |

Model 1, unadjusted; Model 2, adjusted for Gender, Age, BMI; Model 3, adjusted for variables in Model 2 as well as Education, Permanent address, Marital status, Hypertension, Dyslipidemia, Diabetes, Psychiatric problems, Smoking, Alcohol consumption.
