# Supplementary material for: Grip strength and depressive symptoms in Chinese middle-aged and older adults: the mediating effects of cognitive function
Source: Front Aging Neurosci. 2024 Oct 9;16:1455546. doi: 10.3389/fnagi.2024.1455546 (PMC11497465; doi:10.3389/fnagi.2024.1455546)
Supplement: Supplementary file 3 [file Table_3.docx]

**Table S3: The moderated mediating effect of grip strength class on depression by cognitive function in Subgroup Analyses**

| Subgroup | Mediator | Total effect | | Indirect effect | | Direct effect | | Proportion mediated, % (95% CI) |
| --- | --- | --- | --- | --- | --- | --- | --- | --- |
|  |  | Coefficient (95% CI) | P value | Coefficient (95% CI) | P value | Coefficient (95% CI) | P value |  |
| Female | drawing | -0.139 (-0.206, -0.078) | <0.001 | 0.002 (-0.002, 0.006) | 0.196 | -0.141 (-0.209, -0.082) | <0.001 | -1.4 (-5.4, 1.2) |
|  | memory | -0.139 (-0.205, -0.078) | <0.001 | -0.002 (-0.007, 0.003) | 0.492 | -0.137 (-0.205, -0.076) | <0.001 | 1.5 (-2.5, 6.7) |
|  | orientation | -0.139 (-0.206, -0.077) | <0.001 | -0.002 (-0.005, 0.001) | 0.184 | -0.137 (-0.204, -0.075) | <0.001 | 1.2 (-0.5, 4.8) |
|  | computation | -0.139 (-0.206, -0.078) | <0.001 | -0.002 (-0.006, 0.001) | 0.160 | -0.136 (-0.203, -0.076) | <0.001 | 1.6 (-0.8, 5.5) |
|  | Cognitive function | -0.139 (-0.206, -0.078) | <0.001 | -0.004 (-0.011, 0.002) | 0.180 | -0.135 (-0.202, -0.075) | <0.001 | 2.8 (-1.7, 9.5) |
| Male | drawing | -0.055 (-0.096, -0.012) | 0.012 | -0.003 (-0.006, -0.000) | 0.040 | -0.052 (-0.094, -0.010) | 0.020 | 5.1 (0.2, 19.8) |
|  | memory | -0.057 (-0.098, -0.015) | 0.008 | -0.007 (-0.012, -0.003) | <0.001 | -0.049 (-0.088, -0.008) | 0.036 | 13.0 (4.6, 45.9) |
|  | orientation | -0.056 (-0.098, -0.013) | 0.012 | -0.005 (-0.009, -0.002) | <0.001 | -0.050 (-0.092, -0.008) | 0.028 | 9.2 (2.3, 39.2) |
|  | computation | -0.055 (-0.097, -0.013) | 0.012 | -0.006 (-0.011, -0.001) | 0.016 | -0.049 (-0.090, -0.006) | 0.028 | 10.6 (2.4, 44.6) |
|  | Cognitive function | -0.056 (-0.098, -0.014) | 0.008 | -0.013 (-0.020, -0.007) | <0.001 | -0.043 (-0.082, -0.000) | 0.048 | 23.8 (11.2, 85.3) |
| 60 years old and above | drawing | -0.134 (-0.197, -0.065) | <0.001 | -0.002 (-0.007, 0.001) | 0.216 | -0.132 (-0.196, -0.061) | <0.001 | 1.8 (-1.3, 6.4) |
|  | memory | -0.134 (-0.198, -0.065) | <0.001 | -0.003 (-0.010, 0.003) | 0.256 | -0.131 (-0.193, -0.061) | <0.001 | 2.3 (-2.2, 9.4) |
|  | orientation | -0.134 (-0.198, -0.064) | <0.001 | -0.002 (-0.007, 0.002) | 0.324 | -0.132 (-0.196, -0.062) | <0.001 | 1.4 (-1.3, 5.7) |
|  | computation | -0.133 (-0.197, -0.064) | <0.001 | -0.004 (-0.008, 0.000) | 0.060 | -0.129 (-0.192, -0.059) | <0.001 | 2.9 (-0.0, 8.1) |
|  | Cognitive function | -0.134 (-0.198, -0.065) | <0.001 | -0.006 (-0.015, 0.000) | 0.060 | -0.127 (-0.188, -0.058) | <0.001 | 4.9 (-0.1, 14.7) |
| 60 years and over. | drawing | -0.064 (-0.103, -0.021) | <0.001 | -0.003 (-0.007, -0.000) | 0.040 | -0.061 (-0.098, -0.020) | <0.001 | 4.8 (0.3, 16.3) |
|  | memory | -0.064 (-0.103, -0.022) | <0.001 | -0.010 (-0.015, -0.005) | <0.001 | -0.054 (-0.093, -0.012) | 0.008 | 15.4 (7.0, 44.0) |
|  | orientation | -0.063 (-0.103, -0.021) | <0.001 | -0.004 (-0.009, -0.000) | 0.028 | -0.059 (-0.098, -0.017) | <0.001 | 6.7 (0.6, 27.0) |
|  | computation | -0.063 (-0.103, -0.021) | <0.001 | -0.006 (-0.011, -0.002) | <0.001 | -0.057 (-0.096, -0.016) | <0.001 | 9.6 (2.7, 26.6) |
|  | Cognitive function | -0.064 (-0.103, -0.022) | <0.001 | -0.015 (-0.022, -0.009) | <0.001 | -0.049 (-0.088, -0.008) | 0.016 | 23.2 (11.8, 65.9) |
| The mediation analyses were adjusted for Age, BMI, Gender, Education, Permanent address, Marital status, Hypertension, Dyslipidemia, Diabetes, Smoking and Alcohol consumption, | | | | | | | | |
